# Abnormal Sensorimotor Integration in Adults Who Stutter: A Behavioral Study by Adaptation of Delayed Auditory Feedback

**DOI:** 10.3389/fpsyg.2019.02440

**Published:** 2019-10-31

**Authors:** Daichi Iimura, Nobuhiko Asakura, Takafumi Sasaoka, Toshio Inui

**Affiliations:** ^1^Graduate School of Comprehensive Human Sciences, University of Tsukuba, Ibaraki, Japan; ^2^Japan Society for the Promotion of Science, Tokyo, Japan; ^3^Domo-Work (Specified Nonprofit Corporation), Tokyo, Japan; ^4^Center for Mathematical Modeling and Data Science, Osaka University, Osaka, Japan; ^5^Brain, Mind and KANSEI Sciences Research Center, Hiroshima University, Hiroshima, Japan; ^6^Department of Psychology, Otemon Gakuin University, Osaka, Japan

**Keywords:** stuttering, sensorimotor integration, auditory feedback, internal prediction, sensory adaptation

## Abstract

Stuttering is a fluency disorder, partially alleviated during altered auditory feedback, suggesting abnormal sensorimotor integration in adults who stutter (AWS). As weighting of multiple integrating-information sources would be decided based on their reliabilities, the use of external (auditory feedback) and internal information (prediction of sensory consequences) could correlate with speech processing. We hypothesized that abnormal auditory-feedback processing in AWS could be related to decrease in internal processing precision. We used a perceptual-adaptation experiment of delayed auditory feedback (DAF) to verify the hypothesis. Seventeen AWS and 17 adults who do not stutter (ANS) were required to say “ah” and judge the simultaneity between their motor sensations and vocal sounds in each stimulus onset asynchrony (SOA) (0, 25, 50, 75, 100, 125, or 150 ms) after inducing adaptation of DAF (three conditions with 0-, 66-, or 133-ms delay). While no adaptation occurred during the 0 ms condition, perceptual change in simultaneity judgment (adaptation effect) occurred during the 66 and 133 ms conditions. The simultaneity judgments following exposure in each SOA were fitted to the psychometric function in each condition for the AWS and ANS groups. We calculated the μ (signifying the point of subjective simultaneity and adaptation-effect degree) and σ (signifying the detecting precision) of each function and analyzed them by parametric analyses. For the μ, participant groups and adaptation conditions showed a significant interaction; the adaptation effect was greater in the AWS than in the ANS group. Additionally, the μ and σ were only positively correlated in the AWS group. The point of subjective simultaneity for auditory delay by inducing DAF was higher in AWS than in ANS, indicating that perception of simultaneity in AWS was influenced by DAF to a greater extent. Moreover, the significant positive correlation between the μ and σ in AWS showed that the more imprecise the internal auditory processing, the more AWS relied on auditory feedback. It is suggested that the reliability of internal information differed within the AWS group, and AWS with reduced internal reliability appeared to compensate by relying to a great extent on auditory feedback information.

## Introduction

Developmental stuttering is a fluency disorder characterized by frequent word or part-word repetition, prolongation, and silent blocks that disrupt the rhythmic flow of speech, especially in the initial parts of utterances (e.g., Van Riper, 1982; Guitar, 2018). An approximate 1% of the adult population and 5% of children stutter, typically starting at 3–5 years of age (e.g., Månsson, 2000; Yairi and Ambrose, 2013). Although the disorder involves brain anatomical and functional abnormalities (e.g., Fox et al., 1996; Etchell et al., 2018), physiological abnormality (e.g., Hutchinson and Watkin, 1976), and dysfunction of sensorimotor speech processing (e.g., Kalinowski et al., 1996), the underlying mechanism remains unclear. Classically, sensorimotor speech deficits in AWS are considered a source of stuttering (e.g., Stromsta, 1972; Neilson and Neilson, 1987; Max et al., 2004; Bloodstein and Ratner, 2008).

The sensorimotor integration of speech is an essential process to realize precise and rapid speech movement. That is, there is an interaction between speech motor control and processing of auditory feedback (note that we focus on auditory feedback processing, although somatosensory or proprioceptive feedback is also used in speech production). When we produce speech and the articulator moves, feedforward and feedback motor control work in parallel. In feedforward control, motor commands are prepared before the onset of movement and then issued to the musculature for articulation without any modification (Kawato, 1999; Max et al., 2004). However, environmental noise or articulator perturbation disturb precise sensory feedback, and as a result, mismatch occurs between the expected and produced auditory signal. Feedback control then works to repair the movement. Efference copies that predict the desired sensory consequences are generated in brain networks in parallel with the motor commands. By continuous comparison of the internal prediction with sensory afferent information, and their integration with the comparator, we can monitor the movement and update motor commands to minimize the mismatch between internal prediction and sensory feedback.

When we integrate external and internal information, their weighting is decided based on their precision or reliability (Ernst and Bülthoff, 2004; Körding and Wolpert, 2004; Moore and Fletcher, 2012). To achieve robust perception, we combine and integrate multiple sources of sensory information (Ernst and Bülthoff, 2004). Relying on external signals (e.g., auditory feedback) is enhanced when the reliability of internal signals (that is, noisy and imprecise internal prediction) is reduced.

Previous behavioral and neurophysiological findings suggest that there is abnormal sensorimotor (e.g., auditory to speech) integration in AWS (e.g., Neilson and Neilson, 1987; Max et al., 2004; Hickok et al., 2011; Daliri et al., 2018). Although we recognize that we could not identify the specific factors that cause stuttering because stuttering is a multi-factored disorder (e.g., Smith and Weber, 2017), we propose a hypothesis that the source of stuttering involves dysfunction of auditory feedback. This is supported by the notion that altered auditory feedback (delayed auditory feedback, frequency altered feedback, and masking auditory feedback) has the temporal effect of alleviating stuttering (e.g., Kalinowski et al., 1993; Lincoln et al., 2006). The low incidence rate of stuttering among deaf persons, who cannot use auditory feedback, was also reported (Montgomery and Fitch, 1988; Bloodstein and Ratner, 2008). Neuroimaging studies have shown atypical brain activity specifically in auditory-related areas in individuals who stutter (see for review: Brown et al., 2005; Budde et al., 2014; Belyk et al., 2017; Etchell et al., 2018). These studies indicated that stuttering disfluency is involved in abnormal sensorimotor integration.

The degree to which AWS use auditory feedback is under debate. Some previous studies have suggested that there is feedback-dominant speech motor control (overreliance on auditory feedback) in AWS (Fukawa et al., 1988; De Nil et al., 2001; Civier et al., 2010). In addition to altered auditory feedback, several conditions such as shadowing (Healey and Howe, 1987), choral speech (Kalinowski and Saltuklaroglu, 2003), and dual tasks (Arends et al., 1988; Bajaj, 2007) reportedly enhance speech fluency in AWS. These conditions could serve to divert speech control from feedback-based motor control, resulting in speech-fluency enhancement (Bloodstein and Ratner, 2008). Fukawa et al. (1988) found that AWS exhibit greater susceptibility to DAF than do ANS and therefore proposed that AWS control speech cognitively (feedback-based) rather than automatically (feedforward-based). De Nil et al. (2001) found greater cerebellar activation in AWS compared to ANS and contested that movement in AWS may be less automatic and more dependent on sensory or motor monitoring. The directions into velocities of articulators model, which is a neural network model of speech production, simulates stuttering based on the dominance of feedback control in speech (Guenther et al., 2006; Civier et al., 2010; Tourville and Guenther, 2011). Unlike ANS, which must rely primarily on feedforward control, AWS produce speech with a motor strategy that relies heavily on auditory feedback control because of impaired readout of feedforward control (Civier et al., 2010). In contrast, recent studies have shown that the corrective motor responses to compensate for unexpected auditory perturbations are smaller in AWS than in ANS (Cai et al., 2012, 2014; Loucks and De Nil, 2012; Daliri et al., 2018). Electrophysiological studies have also reported reduced modulation of auditory evoked potentials that decreased the preparation of the auditory system during speech planning in AWS (Daliri and Max, 2015a, b; Mock et al., 2015). The inefficiency in auditory-motor integration (or sensory-motor integration in general) may be a result of reduced reliance on auditory feedback (Loucks and De Nil, 2006; Daliri et al., 2013).

We emphasize again that sensory integration is weighted according to the reliability of the internal prediction and outer sensory feedback (Ernst and Bülthoff, 2004; Körding and Wolpert, 2004; Moore and Fletcher, 2012). The degree to be applied is determined based on this weighting. Although not sufficiently documented, it is suggested that the internal action-related prediction signal is noisy and imprecise in AWS. Hickok et al. (2011) proposed a theoretical model of speech processing and argued that imprecise mapping between the sensory and motor systems could be a source of dysfunction in stuttering. They argued that in AWS, because of anatomical asymmetry in the planum temporale (Foundas et al., 2004), which could contain the area of sensorimotor transformation, the mapping between the internal model of the vocal tract and the sensory system is noisy, resulting in increased variance of the mapping function. A corticoanatomical disconnection involving the region of the prediction signal was also observed (Sommer et al., 2002). Max et al. (2004) also hypothesized that stuttering results from unstable or insufficiently activated internal models based on the schematic representation of a speech control model. These notions support the hypothesis of the noisy internal prediction signal or mapping that may result in less reliable output from the comparator.

In sum, internal abnormal processing and maladaptive responses could underlie the deficits of auditory feedback processing in AWS. The degree of using auditory feedback should be determined by the prediction signal’s reliability. In other words, to achieve precise auditory perception, the relative weighting toward auditory feedback could be associated with that of internal processing. However, the imprecision of the internal prediction signal or mapping and the relationship between the degrees of internal precision and external auditory feedback reliance remain unclear.

We then hypothesized that deficits in integration of auditory feedback in AWS are related to decrease in the reliability of internal processing and simultaneously investigated three variables as independent factors: reliance on auditory feedback, precision of internal processing, and the correlation between them. We performed a behavioral experiment using adaptation of altered auditory feedback. Adaptation is the process of adjusting one’s perception or actions to new situations (e.g., the gradual variations in bone growth or muscle-mass increases or the change in motor dynamics over a shorter timescale) (Shadmehr et al., 2010; Wolpert et al., 2011), such as visuo-motor (Synofzik et al., 2010), auditory-motor (Daliri and Max, 2018; Daliri et al., 2018), or auditory-perception (Yamamoto and Kawabata, 2011, 2014) adaptation. We could obtain a benefit by using altered auditory feedback because this experimental condition does not change the somatosensory feedback (e.g., motor perturbation) and we could exclude the somatosensory effect. In line with a previous DAF adaptation experiment (Yamamoto and Kawabata, 2011, 2014), we used a simultaneity judgment task with AWS and ANS between voice onset and perception of auditory feedback after inducing a voice lag adaptation. By investigating the degree of perceptual changes in simultaneity judgment (i.e., adaptation effects), it is possible to show the degree to which individuals rely on sensory feedback information (Synofzik et al., 2010). If the adaptation effect is small, it would denote that sensory information is used to a limited degree. In contrast, a greater adaptation effect would denote that individuals rely more on sensory feedback, thus updating the prediction signal considerably. The consistency of the simultaneity judgment (i.e., participant judgment between the auditory feedback and internal sensory prediction converted from the efferent motor signal generated in parallel with the speech command) reflects the precision of the internal signal itself, which predicts auditory consequences or integration processing, as sensory information is not changed within one experimental condition. More precise internal processing could reduce the variance of judgment. More importantly, the relationship between the adaptation-effect degree and the variability of the judgment reflects the relationship between reliance on auditory feedback and precision of the prediction signal. In the experimental paradigm of DAF adaptation, we could investigate the specific aspect of sensorimotor integration, i.e., the temporal perception of auditory feedback in AWS.

## Materials and Methods

### Ethics Statement

The ethics committee of the Unit for the Integrated Studies of the Human Mind, Kyoto University approved the experimental procedures in advance, and the experiment was conducted in accordance with the principles of the Declaration of Helsinki. Prior to the experiment, we obtained written informed consent from each participant after information was provided regarding the study purpose, methodology, risks, duration of the experiment, handling of personal information, benefit of the study’s result, rights to withdraw, and voluntary participation.

### Participants

The AWS group consisted of 17 adults (five women, one left-handed) aged 19–30 years (mean age and standard deviation, 23.7 ± 3.3 years), and the ANS group consisted of 17 adults (five women, one left-handed) aged 20–29 years (23.5 ± 2.7 years). The two groups were matched for age (*t*(32) = 0.23, *p* = 0.82; unpaired *t*-test), sex, and their dominant hand. All participants were native speakers of Japanese. No participant had history of neurological problems or speech or language problems, except for stuttering in the AWS group. Their hearing was normal, to the extent that all participants could normally participate in conversation.

### Experimental Settings and Stimuli

**FIGURE 1:**
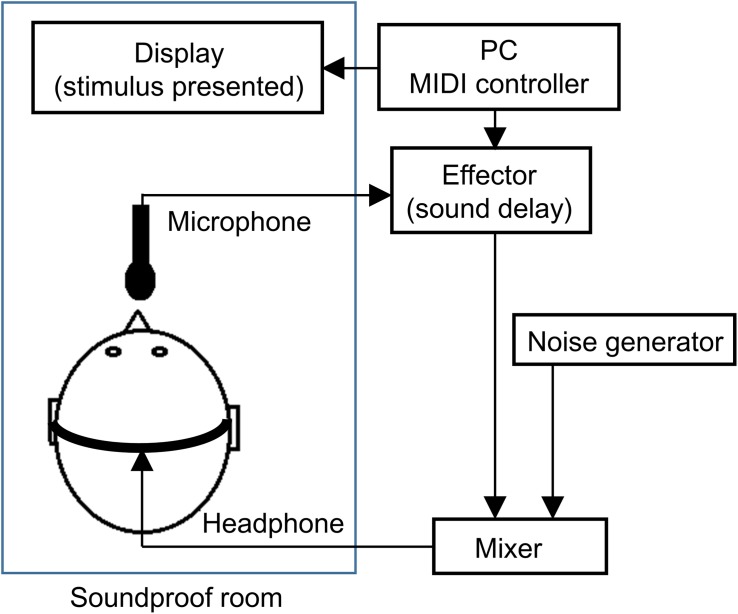
Schematic diagram of the experimental system.

The experiment was conducted individually in a closed, soundproof room. The participants remained in the soundproof room alone and they were seated in a chair. The experimental setting is shown in Figure 1. A display monitor (LL-T174-B, SHARP, Osaka, Japan) was placed on a desk in front of the participant. Participants were required to look at the display wearing headphones (HP-RX500, JVC, Kanagawa, Japan). Participant voices were recorded by a microphone (ECM-G5M, SONY, Tokyo, Japan) and fed back in real-time on the headphones through an auditory effector (MX300, LEXICON, Waltham, MA, United States) and audio mixer (802VLZ4, MACKIE, Woodinville, WA, United States). The auditory effector was connected to the microphone in order to produce auditory delays between voice onset and voice perception. Delay times were manipulated using MATLAB (2012a, MathWorks, Natick, MA, United States) on a Windows PC (PRECISION T1600, DELL, Tokyo, Japan), and MIDI controller on the PC send a command signal to the effector. Pink noise at a 90-dB sound pressure level was constantly presented through the headphones during each block for the purpose of masking the participants’ voice via air duct sound and disrupting any potential additional auditory cues other than the feedback from the headphones. The auditory feedback of the participants’ voice and masking noise were composed through an audio mixer before being presented through the headphones. We confirmed that the participants could hear their voice through the headphones.

### Procedure

**FIGURE 2:**
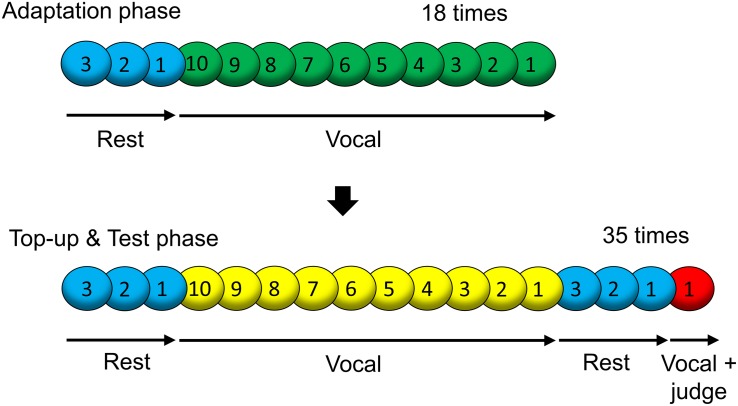
Sequence of visual cues presented on the display in one block. One block consisted of the “Adaptation phase” and “Top-up and Test phase (maintaining adaptation and response).”

A visual cue with a blue, green, yellow, or red circle was successively presented at the center of the display for 1 s. Numbers on the colored circles were presented as an instructional countdown (i.e., 3, 2, 1, as shown in Figure 2).

The experimental design and procedure were according to previous studies on DAF adaptation (Yamamoto and Kawabata, 2011, 2014). Figure 2 presents the time course of one block. One block contained an “Adaptation phase (inducing adaptation)” and a “Top-up and Test phase (maintaining adaptation and response).” In the “Adaptation phase,” three blue cues and 10 green cues were successively presented, and this loop was repeated 18 times. Blue cues denoted rest. When the green cue was presented, participants were required to say “ah.” Since 10 green cues were presented, the participants said “ah” 10 times at intervals of 1 s in one loop. The participants’ voices were presented via the headphones with a constant delay in a block (the adapted SOA: 0, 66, or 133 ms), inducing lag adaptation. In the “Top-up and Test phase,” three blue cues, 10 yellow cues, three blue cues, and a red cue were successively presented, and this loop was repeated 35 times. The yellow cues played the role of the green cues (the adapted SOA was also the same). With the red cue, the participants were instructed to say “ah” and judge the simultaneity between their motor sensations and vocal sounds at that time (test SOA of 0, 25, 50, 75, 100, 125, or 150 ms.). The test SOAs in the 35 trials were in random order; every SOA occurred five times in each block. The judgment was performed by pressing a keyboard key (left arrow or right arrow) located in front of the participants’ right hand. The left arrow key was labeled “simultaneous” and the right arrow key was labeled “delayed.”

Every participant completed six blocks in the entire experiment; two blocks in each condition (adapted SOA of 0, 66, or 133 ms). Thus, there were 70 judgment trials, and each of the seven SOAs was repeated 10 times. The six blocks were divided across 2 days and counterbalanced for order across participants. Between blocks, there was 5 min break for rest.

For the purpose of assessing stuttering severity in AWS, a reading task (the participants read a passage of approximately 550 syllables) and a speaking task (the participants produced spontaneous speech for 1 min) were administered to the AWS group before the experiment. All speech samples were video recorded with a video camera (HDR-XR550V, SONY) and used by two qualified and trained speech therapists to assess the stuttering severity of the AWS using the SSI-3 (Riley, 1972). To ensure the measurement reliability of the SSI score ratings, stuttering disfluencies were recalculated by the first speech therapist for 10% of the sample selected at random (intra-rater reliability), and inter-rater reliability was determined by a second speech therapist calculating the stuttering scores. Then, an intra-class correlation coefficient analysis was conducted.

### Data Analysis

The individual proportion of “delayed” responses in each test SOA (0, 25, 50, 75, 100, 125, or 150 ms) was calculated as a function of the SOA for each condition (adapted SOAs of 0, 66, or 133 ms). In total, each participant completed six blocks (three conditions), and as a result, there were 10 judgments in each test SOA of each adapted SOA condition; these trials were then entered into the analysis. A cumulative Gaussian psychometric function was then fitted by Probit analysis with/without a lapse parameter, and the function was selected based on Akaike’s Information Criteria (Akaike, 1974). We also calculated the deviance of this psychometrical model relative to the saturated model (Kingdom and Prins, 2010), and it did not show a significant improvement in fit (*p* > 0.05) compared to the saturated model. This confirmed the goodness of fit in each participant in the three conditions. Fitted psychometric functions have two parameters; mean (μ: the interpolated 50% crossover point) and standard deviation (σ: represents the slope of the psychometric function). The μ represents the point of subjective simultaneity (PSS) and adaptation-effect degree, and the σ represents the precision of simultaneous judgment. The μ and σ were calculated in each condition for all participants and separately analyzed by a 2 (Group: AWS vs. ANS) × 3 (Adapted SOA: 0 vs. 66 vs. 133 ms) mixed-design analysis of variance with Shaffer’s multiple comparisons. The μ in the adapted SOA 66- or 133-ms condition denotes the susceptibility of the disruption by DAF, and the 0-ms condition was considered the baseline. The σ denotes participant judgment consistency. We regarded the adapted SOA 0-ms condition as the control condition for comparisons. Finally, to examine the relationship between the adaptation-effect degree and consistency of judgment, Pearson’s correlation analysis was performed between the Δμ (difference between two of the three conditions) and σ within each group. The difference of μ between two of the three conditions denotes the degree of the adaptation effect (i.e., a small difference between e.g., the 0- and 66-ms conditions denotes that adaptation was minor and thus that adaptation of DAF had limited influence, and vice versa). We combined all Δμ and σ condition combinations; i.e., Δμ was the μ of the “66–0 ms conditions,” “133–0 ms conditions,” and “133–66 ms conditions,” and σ was the σ of the 0-, 66-, and 133-ms conditions.

## Results

**FIGURE 3:**
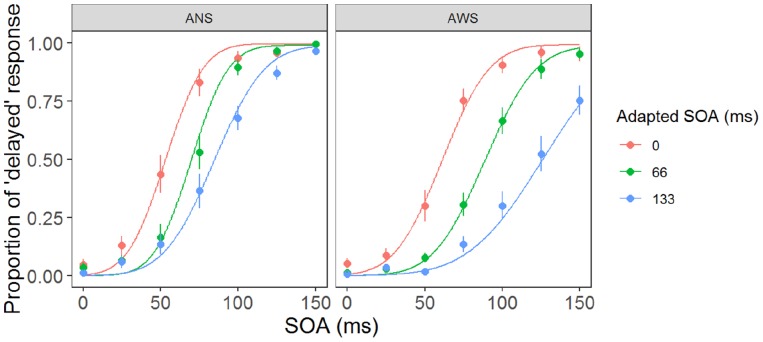
Psychometric function in the three adaptation conditions. Each function was fitted using the parameters of mean μ and σ of the AWS and ANS groups. The plots and error bars represent the means and standard errors (SEs, respectively) of each participant’s psychometric function. ANS, adults who do not stutter; AWS, adults who stutter.

**FIGURE 4:**
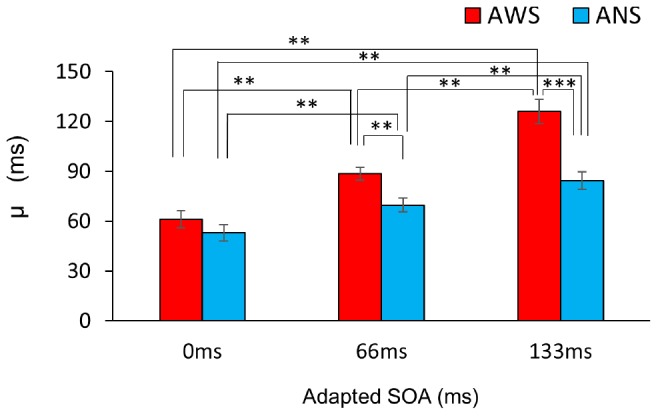
Comparison of the mean μ of the psychometric function. Red bars indicate AWS and blue bars indicate ANS. Error bars represent standard errors (SEs). ^∗∗∗^*p* < 0.001, ^∗∗^*p* < 0.01. Mixed-design analysis of variance. ANS, adults who do not stutter; AWS, adults who stutter.

Figure 3 shows the fitted psychometric function in the three conditions in the AWS and ANS groups. The horizontal axis represents the SOA when participants heard their own voice, and the vertical axis represents their response ratio of correct judgment (except when the SOA was 0 ms, as there was no delay), i.e., the ratio when they responded that their auditory feedback was “delayed.” The μ (Figure 4) showed a significant main effect of group (*F*(1,32) = 128.91, *p* < 0.001, ${\text{η}}_{\text{G}}^{2}$ = 0.469), adapted SOA (*F*(2,64) = 12.09, *p* < 0.01, ${\text{η}}_{\text{G}}^{2}$ = 0.228), and an interaction between them (*F*(2,64) = 16.19, *p* < 0.001, ${\text{η}}_{\text{G}}^{2}$ = 0.100). In the *post hoc* analysis, the μ in the 66-ms condition was significantly higher than that in the 0-ms condition (*t*(16) = 7.61, *p* < 0.01, *d* = 1.443 in AWS; *t*(16) = 4.08, *p* < 0.01, *d* = 0.874 in ANS) and it was significantly higher in the 133-ms condition than in the 0-ms (*t*(16) = 14.33, *p* < 0.01, *d* = 2.477 in AWS; *t*(16) = 6.08, *p* < 0.01, *d* = 1.504 in ANS) and 66-ms (*t*(16) = 8.69, *p* < 0.01, *d* = 1.553 in AWS; *t*(16) = 4.12, *p* < 0.01, *d* = 0.772 in ANS) conditions. When comparing the AWS and ANS groups, significant differences were observed in the 66-ms (*F*(1,32) = 10.96, *p* < 0.01, ${\text{η}}_{\text{G}}^{2}$ = 0.255) and 133-ms (*F*(1,32) = 21.53, *p* < 0.001, ${\text{η}}_{\text{G}}^{2}$ = 0.402) conditions, but not in the 0-ms condition (*F*(1,32) = 1.21, *p* = 0.28, ${\text{η}}_{\text{G}}^{2}$ = 0.037), which indicated that the μ in the 66-ms and 133-ms conditions in the AWS group was significantly higher than that in the ANS group. These results show that when DAF adaptation occurred, the AWS required longer to detect the auditory delay of their voices than did the ANS. In contrast, the lack of significant difference in the 0-ms condition signified that the AWS and ANS groups did not differ in their PSS when there was no adaptation.

**FIGURE 5:**
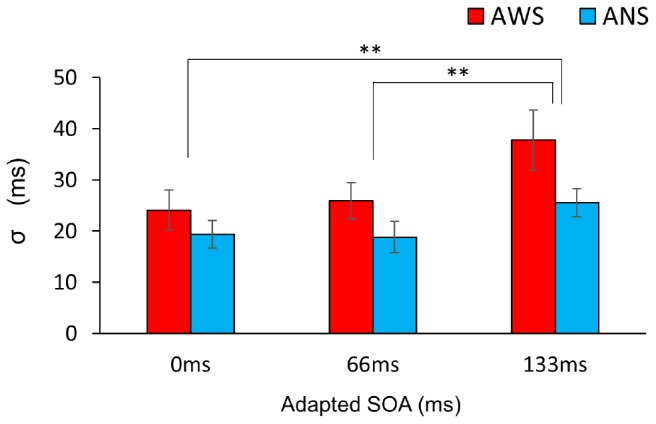
Comparison of the mean σ of the psychometric function. Red bars indicate AWS and blue bars indicate ANS. Error bars represent standard errors (SEs). ^∗∗^*p* < 0.01. Mixed-design analysis of variance. ANS, adults who do not stutter; AWS, adults who stutter.

**FIGURE 6:**
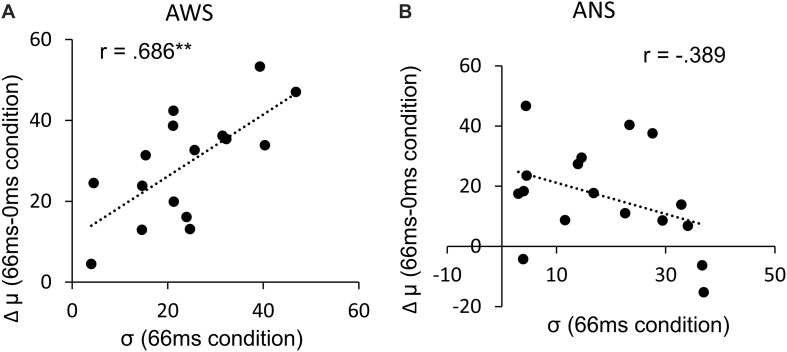
Pearson’s correlation between the Δμ and σ. **(A)** Distribution of AWS with the exception of one outlier (*n* = 16). **(B)** Distribution of ANS (*n* = 17). ^∗∗^*p* < 0.01. ANS, adults who do not stutter; AWS, adults who stutter.

The σ (Figure 5) showed a significant main effect of adapted SOA (*F*(2,64) = 10.95, *p* < 0.001, ${\text{η}}_{\text{G}}^{2}$ = 0.083) but no significant main effect of group (*F*(1,32) = 3.04, *p* = 0.09, ${\text{η}}_{\text{G}}^{2}$ = 0.065) and interaction between them (*F*(2,64) = 1.31, *p* = 0.27, ${\text{η}}_{\text{G}}^{2}$ = 0.011). In the *post hoc* analysis, the σ in the 133-ms condition was significantly higher than that in the 0-ms condition (*t*(32) = 3.71, *p* < 0.01, *d* = 0.588) and in the 66-ms condition (*t*(32) = 4.02, *p* < 0.01, *d* = 0.550) in the within-group comparison. This signifies that there was no difference in prediction precision in the group comparison. Next, analysis of the correlation coefficient between Δμ and σ showed that while no significant correlation between Δμ and σ (*r* = −0.39, *p* = 0.123) was observed in the ANS group, a significant correlation was observed in the AWS group (*r* = 0.69, *p* = 0.003) (Figure 6 as an example among the combinations of Δμ and σ) (Pearson’s correlation analysis, *n* = 16 in the AWS group and *n* = 17 in the ANS group). This correlation tendency in the AWS and ANS groups was observed in most combinations of Δμ and σ (Table 1).

**TABLE 1:** Correlation coefficients in the several conditions of the μ and σ.

**Condition**	**Δμ (66–0 ms)**		**Δμ (133–0 ms)**		**Δμ (133–66 ms)**	
**Group**	**AWS**	**ANS**	**AWS**	**ANS**	**AWS**	**ANS**
σ (0 ms)	0.282	−0.316	0.593^∗^	−0.209	0.655^∗∗^	−0.057
σ (66 ms)	0.686^∗∗^	−0.389	0.563^∗^	−0.393	0.423^†^	−0.126
σ (133 ms)	0.496^†^	−0.028	0.522^∗^	−0.032	0.404^∗^	−0.014

*Between the μ and σ, the Pearson’s correlation coefficient of the AWS and ANS groups with the exception of one Δμ outlier (66–0 ms conditions) in the AWS group. ^∗∗^*p* < 0.01, ^∗^*p* < 0.05, ^†^*p* < 0.1. ANS, adults who do not stutter, AWS, adults who stutter.*

**FIGURE 7:**
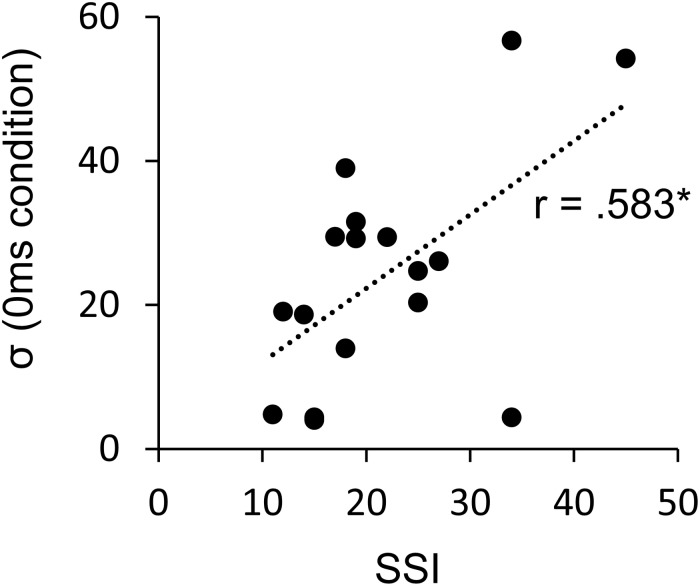
Pearson’s correlation between the σ and the SSI score in AWS (*n* = 17). ^∗^*p* < 0.05. AWS, adults who stutter; SSI, Stuttering Severity Instrument.

The SSI scores of the AWS varied from very mild to very severe (mean = 21.7, SD = 9.1; seven very mild, five mild, three moderate, two severe, one very severe), and we could obtain sufficient reliability of assessment (intra-rater reliability: 0.74, inter-rater reliability: 0.99). While the SSI score and μ did not show a significant correlation, the SSI score and σ were positively significantly correlated (*r* = 0.58, *p* = 0.014) (Figure 7) (Pearson’s correlation analysis, *n* = 17). This suggests the involvement of stuttering severity in the precision of internal prediction.

## Discussion

The purpose of the present study was to investigate speech sensorimotor integration in AWS by adapting the auditory delay of the participants’ voice. The μ of the 0-ms condition did not differ between the AWS and ANS groups, and thus the participants in the two groups could have been equally capable hearing-wise to complete this experiment. Regarding the μ of the psychometric function, both groups were influenced by DAF, with the result of exhibiting a higher PSS for auditory delay induced by DAF adaptation, and this trend was more remarkable in the AWS group than in the ANS group. This signified that by inducing DAF adaptation, the PSS shifted to a higher direction to compensate for perceptual temporal discrepancies in both AWS and ANS. In the case of ANS, the result is consistent with previous findings that showed temporal recalibration of speech after lag adaptation (Yamamoto and Kawabata, 2011, 2014). We contributed with the novel finding that compared to ANS, the simultaneity judgment in AWS is greatly shifted in the direction of exposure delays. This denoted that AWS were influenced by DAF to a greater extent. These maladaptive responses to mismatches between predicted and actual consequences partly supported the notion that AWS rely on auditory feedback to a greater extent. Because AWS rely a greater extent on auditory feedback, the adaptation effect of their simultaneity judgment could be considerably influenced by auditory delay. This is consistent with some previous findings of AWS overreliance on auditory feedback in speech motor control (Fukawa et al., 1988; De Nil et al., 2001; Max et al., 2004; Vasic and Wijnen, 2005; Guenther et al., 2006; Bloodstein and Ratner, 2008; Civier et al., 2010), but some studies have produced opposite results (Loucks and De Nil, 2006; Cai et al., 2012, 2014; Loucks and De Nil, 2012; Daliri et al., 2018). This inconsistency may be interpreted as follows. First, there were some methodological issues, such as differences in the modalities used among studies. The former studies used DAF, MAF, or other fluency-enhancing conditions in their behavioral experiments (Fukawa et al., 1988; Lincoln et al., 2006; Bloodstein and Ratner, 2008) or computed situational studies (Civier et al., 2010). The latter studies mainly focused on the modulated auditory feedback (Cai et al., 2012, 2014; Daliri and Max, 2018; Daliri et al., 2018), somatosensory feedback (Daliri et al., 2013), or auditory evoked potentials in response to probe tones (Daliri and Max, 2015a, b, 2018). Regarding these studies, our scope of investigation was a specific aspect of sensorimotor integration, i.e., the temporal perception of auditory feedback. Thus, the modalities or parameters used in previous studies could have contributed to the observed differences. Given the widespread notion that stuttering is a timing or rhythm disorder (e.g., Alm, 2004; Etchell et al., 2014, 2015; Guitar, 2018) and that Tsakiris et al. (2005) proposed two pathways of efferent signals, timing information (“row” motor command) and other movement descriptors (e.g., kinematic), our study findings could contribute to the mechanism of the sensorimotor timing process of speech. Another reason for the inconsistency between our results and those of some previous studies could be the difference in the adaptation levels used in the respective experiments. Previous studies examined the modulation function of AWS’ speech characteristics that did not usually involve participant awareness, when the auditory feedback was altered (Cai et al., 2012, 2014; Daliri and Max, 2018), treating it as a dependent variable. However, we investigated “perception (judgment)” after auditory adaptation. We have to distinguish these findings and discuss them with caution. Our findings could contribute to the literature in that the sensorimotor system in AWS is affected by auditory feedback distortion to a greater degree than it is in ANS (see Fukawa et al., 1988).

Then, we should speculate on why AWS rely more on auditory feedback. The group difference between AWS and ANS in σ *per se* did not reach significance. Collectively, the precision of the prediction signal in AWS was not different than that in ANS, even when adaptation of DAF occurred. This would signify that AWS did not simply have a sensory deficit in the processing of the temporal information of their voice. Interestingly, we found a significant positive correlation between Δμ and σ only in the AWS group. That is, the more imprecise the internal auditory processing in AWS, the more they rely on auditory feedback. This is consistent with our hypothesis regarding the framework of internal and external integration of information. To increase the reliability of our final perception, we combine different sources based on their precision or reliability (Ernst and Bülthoff, 2004; Moore and Fletcher, 2012). In voluntary action, because internal cues presumably provide highly reliable information, internal prediction would receive higher weighting (Tsakiris et al., 2005) and external cues would receive lower weighting. Conversely, when internal cues are unreliable for some reason (i.e., experimental manipulation to produce noise), external cues carry relatively more weight (e.g., Moore et al., 2009). The result of the present study suggested that the precision of internal processing differed within the AWS group and that AWS who placed less weight to internal information could compensate for this internal issue by placing more weight on external information (auditory feedback).

This resembles the mechanism of positive symptoms in patients with schizophrenia, which involves misattribution of one’s own actions to an external agent. Synofzik et al. (2010) indicated that patients with schizophrenia relied more strongly on the visual feedback of an action rather than on the internal prediction signal. Dysfunction of the dopamine system is implicated in both stuttering and schizophrenia (Alm, 2004; Winterer and Weinberger, 2004; Maguire et al., 2012). Thus, a similar mechanism could be involved in the atypical sensorimotor integration. Previous studies have suggested the involvement of abnormalities in basal ganglion activity or in the dopamine system in AWS (Alm, 2004; Brown et al., 2005). The activity of the basal ganglia would influence the internal timing processing of speech production involving the dopamine projections from the substantia nigra pars compacta to the striatum (Alm, 2004). Dopaminergic neuron firing could code the reliability of signals (Fiorillo et al., 2003). In addition, dysfunction of the dopamine system increases internal noise in patients with schizophrenia (Weinberger et al., 2001; Winterer and Weinberger, 2004; Howes et al., 2012), and could result in overreliance on external information (Synofzik et al., 2010). This could also be the case in stuttering; that is, dopaminergic abnormality could render the internal processing noisy, leading AWS to over-rely on external auditory feedback. Moreover, the present study found that the correlation between stuttering severity and σ was significant; individuals with severe stuttering tended to be less precise in their judgments. Therefore, it is suggested that stuttering severity in AWS is correlated with the precision of the prediction signal. Giraud et al. (2008) reported a correlation between severity of stuttering and activity in the basal ganglia, and the findings of this neuroimaging study further support the relationship between the stuttering severity and imprecise prediction signal observed in the present study.

In contrast, no significant correlation between μ and σ was observed in ANS. This signifies that ANS’s precision of internal processing and reliance on auditory feedback are independent of each other. One interpretation is that because the ANS could control speech movement mainly based on the feedforward signal rather than on the feedback signal (Kawato et al., 1987; Civier et al., 2010), they could place relatively less weight on feedback control. Less weight on feedback control and greater weight on feedforward control inversely signified that the ANS did not need to assign weights to the feedback control system (i.e., both to internal prediction and external sensory information), and this is why the precision of the internal prediction and reliance on auditory feedback in AWS do not influence each other. In other words, the extent to which individuals rely on feedback control during speech movement could determine the degree of correlation between the precision of the prediction signal and reliance on auditory feedback.

The findings of the present study concern the “timing” process of abnormal sensorimotor integration, as we employed DAF. Previous studies also proposed that stuttering is a deficit in brain timing networks (e.g., Alm, 2004; Etchell et al., 2014, 2015; Guitar, 2018). This notion follows from the observation that external stimulation, such as the rhythm produced by a metronome, choral speech, or singing, temporarily alleviates stuttering (Alm, 2004; Saltuklaroglu et al., 2004; Snyder et al., 2009), and the behavioral asynchrony and variability of the task performances that are involved in timing (Hulstijn et al., 1992; Zelaznik et al., 1997; Boutsen et al., 2000). Based on brain activation patterns, it was shown that the external timing networks (induced by the external timing cues) compensate for the internal timing networks (Ingham et al., 2012). As mentioned earlier, an atypical brain activation pattern of the basal ganglia has been reported (Wu et al., 1995, 1997; Braun et al., 1997; Giraud et al., 2008). Taken together, it is suggested that because the timing processing of AWS, e.g., the prediction signal of auditory feedback, is noisy, AWS employ the strategy of relying more on auditory timing information.

We further discuss implications for future studies. We emphasize again that we employed a perceptual DAF adaptation task in this study and investigated the specific processing of sensorimotor integration. As Tsakiris et al. (2005) proposed, the prediction signal could be separated to timing information and other information (processed by the forward predictive model) based on a behavioral self-recognition experiment. We assume that our brain separately encodes timing and other information (e.g., somatosensory feedback, see Civier et al., 2010). There could also be a limitation of measurement validity, as we used temporal perception (judgment) to measure participant auditory prediction. In addition, an experimental limitation was that we could not distinguish the precision of internal processing, neither the prediction signal of sensory consequences nor the mapping of different sources. Furthermore, it remains to be revealed whether this atypical sensorimotor integration is specific to DAF [or the same for other types of adaptation of altered (e.g., frequency) auditory feedback]. It also remains unclear whether lag adaptation affects the consistency of auditory simultaneity judgment. The present study found a significant decrease in the judgment consistency, whereas Yamamoto and Kawabata (2011) did not reveal such an effect. Further studies are needed to address this issue. Additionally, we should mention as a study limitation that this DAF adaptation task used the participant’s own voice as feedback. As we did not adopt a control experiment, it remains unclear whether the result is generalizable to other acoustic stimuli. An additional experiment could clarify the involvement of speech motor processing in the AWS sensorimotor integration deficit. Additionally, it should be noted that our study had a relatively small sample and only enrolled adults and thus, we could not determine whether this atypical sensorimotor integration was inherent or acquired, i.e., the cause or effect of stuttering. It was shown that while the adaptation magnitude of AWS in response to formant perturbation was smaller than that of ANS, this was not true for children who stutter (Daliri et al., 2018). Structural and functional abnormalities in neural networks involved in sensorimotor processes are already present in children who stutter (e.g., Chang and Zhu, 2013; Chang et al., 2015). Insufficiency in the internal model used for sensorimotor control of speech movement is caused by incorrect learning/updating in childhood (Max et al., 2004). Taken together, the abnormal sensorimotor integration in AWS could be the result of dysfunction of motor learning or brain activity in childhood. Although these implications regarding the adaptation modalities and the participants’ age remain to be elucidated, our results could shed light on the sensorimotor integration of auditory feedback in AWS.

## Data Availability Statement

The datasets generated and/or analyzed for this study are not publicly available due to specifications on data availability within the ethics approval. Data are, however, available from the corresponding author upon reasonable request and with the permission of the ethics committee.

## Author Contributions

DI, NA, TS, and TI conceived and designed the experiments. DI performed the experiments, analyzed the data, and contributed to the writing of the manuscript. DI, NA, and TS contributed with the reagents, materials, and analysis tools.

## Conflict of Interest

The authors declare that the research was conducted in the absence of any commercial or financial relationships that could be construed as a potential conflict of interest.

## Abbreviations

ANS: adults who do not stutter
AWS: adults who stutter
DAF: delayed auditory feedback
MAF: masking auditory feedback
SOA: stimulus onset asynchrony
SSI: Stuttering Severity Instrument.

## References

[B1] Akaike H. (1974). A new look at the statistical model identification. *IEEE Trans. Automat. Contr.* 19 716–723. 10.1109/TAC.1974.1100705

[B2] Alm P. A. (2004). Stuttering and the basal ganglia circuits: a critical review of possible relations. *J. Commun. Disord.* 37 325–369. 10.1016/j.jcomdis.2004.03.001 15159193

[B3] Arends N. Povel D. J. Kolk H. (1988). Stuttering as an attentional phenomenon. *J. Fluency Disord.* 13 141–151. 10.1016/0094-730X(88)90035-6

[B4] Bajaj A. (2007). Working memory involvement in stuttering: exploring the evidence and research implications. *J. Fluency Disord.* 32 218–238. 10.1016/j.jfludis.2007.03.002 17825670

[B5] BelykM.KraftS. J.BrownS. (2017). Stuttering as a trait or a state revisited: motor system involvement in persistent developmental stuttering. *Eur. J. Neurosci.* 45 622–624. 10.1111/ejn.13512 28191730

[B6] Bloodstein O. Ratner N. B. (2008). *A Handbook on Stuttering*, 6th Edn New York, NY: Thomson-Delmer.

[B7] Boutsen F. R. Brutten G. J. Watts C. R. (2000). Timing and intensity variability in the metronomic speech of stuttering and nonstuttering speakers. *J. Speech Lang. Hear. Res.* 43 513–520. 10.1044/jslhr.4302.513 10757700

[B8] Braun A. R. Varge M. Stager S. Schulz G. Selbie S. Maisog J. M. (1997). Altered patterns of cerebral activity during speech and language production in developmental stuttering. *Brain* 120 761–784. 10.1093/brain/120.5.761 9183248

[B9] Brown S. Ingham R. Ingham J. C. Laird A. R. Fox P. T. (2005). Stuttered and fluent speech production: an ALE meta-analysis of functional neuroimaging studies. *Hum. Brain Mapp.* 25 105–117. 10.1002/hbm.20140 15846815 PMC6871755

[B10] Budde K. S. Barron D. S. Fox P. T. (2014). Stuttering, induced fluency, and natural fluency: a hierarchical series of activation likelihood estimation meta-analysis. *Brain Lang.* 139 99–107. 10.1016/j.bandl.2014.10.002 25463820 PMC4405378

[B11] Cai S. Beal D. S. Ghosh S. S. Guenther F. H. Perkell J. S. (2014). Impaired timing adjustments in response to time-varying auditory perturbation during connected speech production in persons who stutter. *Brain Lang.* 129 24–29. 10.1016/j.bandl.2014.01.002 24486601 PMC3947674

[B12] Cai S. Beal D. S. Ghosh S. S. Tiede M. K. Guenther F. H. Perkell J. S. (2012). Weak responses to auditory feedback perturbation during articulation in persons who stutter: evidence for abnormal auditory-motor transformation. *PLoS One* 7:e41830. 10.1371/journal.pone.0041830 22911857 PMC3402433

[B13] Chang S. E. Zhu D. C. (2013). Neural network connectivity differences in children who stutter. *Brain* 136 3709–3726. 10.1093/brain/awt275 24131593 PMC3859219

[B14] Chang S. E. Zhu D. C. Choo A. L. Angstadt M. (2015). White matter neuroanatomical differences in young children who stutter. *Brain* 138 694–711. 10.1093/brain/awu400 25619509 PMC4339778

[B15] Civier O. Tasko S. M. Guenther F. H. (2010). Overreliance on auditory feedback may lead to sound/syllable repetitions: simulations of stuttering and fluency-inducing conditions with a neural model of speech production. *J. Fluency Disord.* 35 246–279. 10.1016/j.jfludis.2010.05.002 20831971 PMC2939043

[B16] Daliri A. Max L. (2015a). Electrophysiological evidence for a general auditory prediction deficit in adults who stutter. *Brain Lang.* 150 37–44. 10.1016/j.bandl.2015.08.008 26335995 PMC4663101

[B17] Daliri A. Max L. (2015b). Modulation of auditory processing during speech movement planning is limited in adults who stutter. *Brain Lang.* 143 59–68. 10.1016/j.bandl.2015.03.002 25796060 PMC4380808

[B18] Daliri A. Max L. (2018). Stuttering adults’ lack of pre-speech auditory modulation normalizes when speaking with delayed auditory feedback. *Cortex* 99 55–68. 10.1016/j.cortex.2017.10.019 29169049 PMC5801108

[B19] Daliri A. Prokopenko R. A. Max L. (2013). Afferent and efferent aspects of mandibular sensorimotor control in adults who stutter. *J. Speech Lang. Hear. Res.* 56 1774–1788. 10.1044/1092-4388(2013/12-0134) 23816664 PMC3795963

[B20] Daliri A. Wieland E. A. Cai S. Guenther F. H. Chang S. E. (2018). Auditory-motor adaptation is reduced in adults who stutter but not in children who stutter. *Dev. Sci.* 21:e12521. 10.1111/desc.12521 28256029 PMC5581739

[B21] De Nil L. F. Kroll R. M. Houle S. (2001). Functional neuroimaging of cerebellar activation during single word reading and verb generation in stuttering and nonstuttering adults. *Neurosci. Lett.* 302 77–80. 10.1016/S0304-3940(01)01671-8 11290391

[B22] Ernst M. O. Bülthoff H. H. (2004). Merging the sense into a robust percept. *Trends Cog. Sci.* 8 162–169. 10.1016/j.tics.2004.02.002 15050512

[B23] Etchell A. C. Civier O. Ballard K. J. Sowman P. F. (2018). A systematic literature review of neuroimaging research on developmental stuttering between 1995 and 2016. *J. Fluency Disord.* 55 6–45. 10.1016/j.jfludis.2017.03.007 28778745

[B24] Etchell A. C. Johnson B. W. Sowman P. F. (2014). Behavioral and multimodal neuroimaging evidence for a deficit in brain timing networks in stuttering: a hypothesis and theory. *Front. Hum. Neurosci.* 8:467. 10.3389/fnhum.2014.00467 25009487 PMC4070061

[B25] Etchell A. C. Johnson B. W. Sowman P. F. (2015). Beta oscillations, timing, and stuttering. *Front. Hum. Neurosci.* 8:1036. 10.3389/fnhum.2014.01036 25601832 PMC4283545

[B26] Fiorillo C. D. Tobler P. N. Schultz W. (2003). Discrete coding of reward probability and uncertainty by dopamine neurons. *Science* 299 1898–1902. 10.1126/science.1077349 12649484

[B27] Foundas A. L. Bollich A. M. Feldman J. Corey D. M. Hurley M. Lemen L. C. (2004). Aberrant auditory processing and atypical pla- num temporale in developmental stuttering. *Neurology* 63 1640–1646. 10.1212/01.WNL.0000142993.33158.2A 15534249

[B28] Fox P. T. Ingham R. J. Ingham J. C. Hirsch T. B. Downs J. H. Martin C. (1996). A PET study of the neural systems of stuttering. *Nature* 382:158. 10.1038/382158a0 8700204

[B29] Fukawa T. Yoshioka H. Ozawa E. Yoshida S. (1988). Difference of susceptibility to delayed auditory feedback between stutterers and nonstutterers. *J. Speech Hear. Res.* 31 475–479. 10.1044/jshr.3103.475 3172765

[B30] Giraud A. L. Neumann K. Bachoud-Levi A. C. von Gudenberg A. W. Euler H. Lanfermann H. (2008). Severity of dysfluency correlates with basal ganglia activity in persistent developmental stuttering. *Brain Lang.* 104 190–199. 10.1016/j.bandl.2007.04.005 17531310

[B31] Guenther F. H. Ghosh S. S. Tourville J. A. (2006). Neural modeling and imaging of the cortical interactions underlying syllable production. *Brain Lang.* 96 280–301. 10.1016/j.bandl.2005.06.001 16040108 PMC1473986

[B32] Guitar B. (2018). *Stuttering: An integrated Approach to its Nature and Treatment*, 5th Edn Baltimore: Lippincott Williams and Wilkins.

[B33] Healey E. C. Howe S. W. (1987). Speech shadowing characteristics of stutterers under diotic and dichotic condition. *J. Commun. Disord.* 20 493–506. 10.1016/0021-9924(87)90036-0 3693593

[B34] Hickok G. Houde J. Rong F. (2011). Sensorimotor integration in speech processing: computational basis and neural organization. *Neuron* 69 407–422. 10.1016/j.neuron.2011.01.019 21315253 PMC3057382

[B35] Howes O. D. Kambeitz J. Kim E. Stahl D. Slifstein M. Abi-Dargham A. (2012). The nature of dopamine dysfunction in schizophrenia and what this means for treatment: meta-analysis of imaging studies. *Arch. Gen. Psychiatry* 69 776–786. 10.1001/archgenpsychiatry.2012.169 22474070 PMC3730746

[B36] Hulstijn W. Summers J. J. van Lieshout P. H. Peters H. F. (1992). Timing in finger tapping and speech: a comparison between stutterers and fluent speakers. *Hum. Mov. Sci.* 11 113–124. 10.1016/0167-9457(92)90054-F

[B37] Hutchinson J. M. Watkin K. L. (1976). Jaw mechanics during release of the stuttering moment: some initial observations and interpretations. *J. Commun. Disord.* 9 269–279. 10.1016/0021-9924(76)90017-4 1018054

[B38] Ingham R. J. Grafton S. T. Bothe A. K. Ingham J. C. (2012). Brain activity in adults who stutter: similarities across peaking tasks and correlations with stuttering frequency and speaking rate. *Brain Lang.* 122 11–24. 10.1016/j.bandl.2012.04.002 22564749 PMC3372660

[B39] Kalinowski J. Armson J. Stuart A. Gracco V. L. (1993). Effects of alterations in auditory feedback and speech rate on stuttering frequency. *Lang. Speech* 36 1–16. 10.1177/002383099303600101 8345771

[B40] Kalinowski J. Saltuklaroglu T. (2003). Choral speech: the amelioration of stuttering via imitation and the mirror neuron system. *Neurosci. Behavi. Rev.* 27 339–347. 10.1016/S0149-7634(03)00063-0 12946686

[B41] Kalinowski J. Stuart A. Sark S. Armson J. (1996). Stuttering amelioration at various auditory feedback delays and speech rates. *Eur. J. Disord. Commun.* 31 259–269. 10.3109/13682829609033157 8944848

[B42] Kawato M. (1999). Internal models for motor control and trajectory planning. *Curr. Opin. Neurobiol.* 9 718–727. 10.1016/S0959-4388(99)00028-8 10607637

[B43] Kawato M. Furukawa K. Suzuki R. (1987). A hierarchical neural-network model for control and learning of voluntary movement. *Biol. Cybern.* 57 169–185. 10.1007/BF00364149 3676355

[B44] Kingdom F. A. A. Prins N. (2010). “Goodness-of-Fit,” in *Psychophysics: A Practical Introduction*, ed. Prins N., (London: Academic Press: an imprint of Elsevier), 226–228.

[B45] Körding K. P. Wolpert D. M. (2004). Bayesian integration in sensorimotor learning. *Nature* 427:244. 10.1038/nature02169 14724638

[B46] Lincoln M. Packman A. Onslow M. (2006). Altered auditory feedback and the treatment of stuttering: a review. *J. Fluency Disord.* 31 71–89. 10.1016/j.jfludis.2006.04.001 16750562

[B47] Loucks T. M. De Nil L. F. (2006). Oral kinesthetic deficit in adults who stutter: a target-accuracy study. *J. Mot. Behav.* 38 238–247. 10.3200/JMBR.38.3.238-247 16709563

[B48] Loucks T. M. De Nil L. F. (2012). Oral sensorimotor integration in adults who stutter. *Folia Phoniatr. Logop.* 64 116–121. 10.1159/000338248 22584121

[B49] Maguire G. A. Yeh C. Y. Ito B. S. (2012). Overview of the diagnosis and treatment of stuttering. *J. Exp. Clin. Med.* 4 92–97. 10.1016/j.jecm.2012.02.001

[B50] Månsson H. (2000). Childhood stuttering: incidence and development. *J. Fluency Disord.* 25 47–57. 10.1016/S0094-730X(99)00023-6

[B51] Max L. Guenther F. H. Gracco V. L. Ghoss S. S. Wallace M. E. (2004). Unstable or insufficiently activated internal models and feedback-biased motor control as source of disfluency: a theoretical model of stuttering. *Contemp. Issues Commun. Sci. Disord.* 31 105–122. 10.1044/cicsd_31_s_105

[B52] Mock J. R. Foundas A. L. Golob E. J. (2015). Speech preparation in adults with persistent developmental stuttering. *Brain Lang.* 149 97–105. 10.1016/j.bandl.2015.05.009 26197258 PMC4586364

[B53] Montgomery B. M. Fitch J. L. (1988). The prevalence of stuttering in the hearing-impaired school age population. *J. Speech Hear. Disord.* 53 131–135. 10.1044/jshd.5302.131 3361855

[B54] Moore J. W. Fletcher P. C. (2012). Sense of agency in health and disease: a review of cue integration approaches. *Conscious Cogn* 21 59–68. 10.1016/j.concog.2011.08.010 21920777 PMC3315009

[B55] Moore J. W. Wegner D. M. Haggard P. (2009). Modulating the sense of agency with external cues. *Conscious Cogn.* 18 1056–1064. 10.1016/j.concog.2009.05.004 19515577

[B56] Neilson M. D. Neilson P. D. (1987). Speech motor control and stuttering: a computational model of adaptive sensory- motor processing. *Speech Commun.* 6 325–333. 10.1016/0167-6393(87)90007-0

[B57] Riley G. D. (1972). *Stuttering Severity Instrument*, 3rd Edn Austin: Pro-Ed. 10.1044/jshd.3703.314 5057250

[B58] Saltuklaroglu T. Kalinowski J. Guntupalli V. K. (2004). Towards a common neural substrate in the immediate and effective inhibition of stuttering. *Int. J. Neurosci.* 114 435–450. 10.1080/00207450490422687 15195350

[B59] Shadmehr R. Smith M. A. Krakauer J. W. (2010). Error correction, sensory prediction, and adaptation in motor control. *Annu. Rev. Neurosci.* 33 89–108. 10.1146/annurev-neuro-060909-153135 20367317

[B60] Smith A. Weber C. (2017). How stuttering develops: the multifactorial dynamic pathways theory. *J. Speech Lang. Hear. Res.* 60 2483–2505. 10.1044/2017_JSLHR-S-16-0343 28837728 PMC5831617

[B61] Snyder G. J. Hough M. S. Blanchet P. Ivy L. J. Waddell D. (2009). The effects of self-generated synchronous and asynchronous visual speech feedback on overt stuttering frequency. *J. Commun. Disord.* 42 235–244. 10.1016/j.jcomdis.2009.02.002 19304293

[B62] Sommer M. Koch M. A. Paulus W. Weiller C. Buchel C. (2002). Disconnection of speech-relevant brain areas in persistent developmental stuttering. *Lancet* 360 380–383. 10.1016/S0140-6736(02)09610-1 12241779

[B63] Stromsta C. (1972). Internal phase disparity of stutterers and nonstutterers. *J. Speech Hear. Res.* 15 771–780. 10.1044/jshr.1504.771 4652398

[B64] Synofzik M. Their P. Leube D. T. Schlotterbeck P. Linder A. (2010). Misattributions of agency in schizophrenia are based on imprecise predictions about the sensory consequences of one’s actions. *Brain* 133 262–271. 10.1093/brain/awp291 19995870

[B65] Tourville J. A. Guenther F. H. (2011). The DIVA model: a neural theory of speech acquisition and production. *Lang. Cogn. Process.* 26 952–981. 10.1080/01690960903498424 23667281 PMC3650855

[B66] Tsakiris M. Haggard P. Franck N. Mainy N. Sirigu A. (2005). A specific role for efferent information in self-recognition. *Cognition* 96 215–231. 10.1016/j.cognition.2004.08.002 15996559

[B67] Van Riper C. (1982). *The Nature of Stuttering*, 2nd Edn Englewood Cliffs, NJ: Prentice-Hall.

[B68] Vasic N. Wijnen F. (2005). “Stuttering as a monitoring deficit,” in *Phonological Encoding and Monitoring in Normal and Pathological Speech*, eds Hartsuiker R. J. Bastiaanse R. Postma A. Wijnen F., (Hove: Psychology Press), 226–247.

[B69] Weinberger D. R. Egan M. F. Bertolino A. Callicott J. H. Mattay V. S. Lipska B. K. (2001). Prefrontal neurons and the genetics of schizophrenia. *Biol. Psychiatry* 50 825–844. 10.1016/S0006-3223(01)01252-5 11743939

[B70] Winterer G. Weinberger D. R. (2004). Genes, dopamine and cortical signal-to-noise ratio in schizophrenia. *Trends Neurosci.* 27 683–690. 10.1016/j.tins.2004.08.002 15474169

[B71] Wolpert D. M. Diedrichsen J. Flanangan J. R. (2011). Principles of sensorimotor learning. *Nat. Rev. Neurosci.* 12 739–751. 10.1038/nrn3112 22033537

[B72] Wu J. C. Maguire G. Riley G. Fallon J. LaCasse L. Chin S. (1995). A positron emission tomography [18F] deoxyglucose study of developmental stuttering. *Neuroreport* 6 501–505. 10.1097/00001756-199502000-00024 7766852

[B73] Wu J. C. Maguire G. Riley G. Lee A. Keator D. Tang C. (1997). Increased dopamine activity associated with stuttering. *Neuroreport* 8 767–770. 10.1097/00001756-199702100-00037 9106763

[B74] Yairi E. Ambrose N. (2013). Epidemiology of stuttering: 21st century advances. *J. Fluency Disord.* 38 66–87. 10.1016/j.jfludis.2012.11.002 23773662 PMC3687212

[B75] Yamamoto K. Kawabata H. (2011). Temporal recalibration in vocalization induced by adaptation of delayed auditory feedback. *PLoS One* 6:e29414. 10.1371/journal.pone.0029414 22216275 PMC3245272

[B76] Yamamoto K. Kawabata H. (2014). Adaptation to delayed auditory feedback induces the temporal recalibration effect in both speech perception and production. *Exp. Brain Res.* 232 3707–3718. 10.1007/s00221-014-4055-1 25106757

[B77] Zelaznik H. N. Smith A. Franz E. Ho M. (1997). Differences in bimanual coordination associated with stuttering. *Acta Psychol.* 96 229–243. 10.1016/S0001-6918(97)00014-0 9434590

